# Designing an efficient organic–inorganic hybrid nanocomposite for simultaneous oxidative/adsorptive desulfurization of model and real fuel oils

**DOI:** 10.1038/s41598-023-42392-8

**Published:** 2023-09-13

**Authors:** Mina Sadrara, Mohammadreza Khanmohammadi Khorrami

**Affiliations:** Chemistry Department, Faculty of Science, Imam Khomeini International University, Qazvin, Iran

**Keywords:** Chemistry, Materials science

## Abstract

In this study, an efficient organic–inorganic hybrid nanocomposite was designed for deep oxidative/adsorptive removal of dibenzothiophene (DBT) from model and real fuel oils employing surface molecularly imprinted polymer (SMIP) and mesoporous silica nanoparticles (MSNs). On the surface of silanol-functionalized MCM-48-HPW prepared at different 12-tungstophosphoric acid (HPW wt%) as the oxidation catalyst, an imprinted polymethacrylic acid polymer (PMAA) as a selective adsorbent of DBT was formed using different amounts of DBT template. Then, various oxidant/sulfur molar ratios were applied during the desulfurization reactions according to the central composite design (CCD). The successful synthesis of the optimum SMIP-PMAA@MCM-48-HPW nanocomposite was confirmed by FTIR, XRD, N_2_-adsorption, SEM, TEM, TGA, and NMR techniques. The desulfurization percentage of the model oil reached 98.54% under the optimum conditions, and the catalyst percentage was found to be the most significant parameter for desulfurization efficiency. Comparison experiments showed that the combined role of oxidation and adsorption had an extensive impact on desulfurization efficiency. Under the optimized conditions, 96% DBT from gasoline was removed by the optimum nanocomposite. The optimum nanocomposite showed good stability and could be reused five times without a remarkable decrease in the desulfurization ability.

## Introduction

Combustion of fuels with high sulfur content has negative effects on the atmospheric environment and human health^1^. Dibenzothiophene (DBT) and its derivatives are major constituents of organosulfur compounds in fossil fuels, which contribute significantly to air pollution. Most countries are moving toward more stringent regulations to reduce the allowable amount of sulfur compounds in fossil fuels to less than 10 mg/L. So, new and efficient methods for the desulfurization of fossil fuels are required. Among the various desulfurization techniques, catalytic oxidative desulfurization (ODS) is a potentially effective technique for deep desulfurization of fuel oils due to mild conditions^2^. 12-tungstophosphoric acid (HPW) as a Keggin-type polyoxometalate (POM) has proved to be a very active and selective oxidation catalyst. However, bulk POMs suffer from some drawbacks, such as low specific surface area (< 10 m^2^g^−1^), low porosity, weak thermal stability, and high solubility in polar media, which reduce access to the active sites and increase separation problems^3^. To overcome these disadvantages, many strategies have been developed for immobilizing POMs within suitable solid supports, such as mesoporous SiO_2_, Al_2_O_3_, ZrO_2_, TiO_2_ and activated carbon to obtain active and easily reusable catalysts^4,5^. Among them, HPW supported on mesoporous silica nanoparticles (MSNs) has been widely used in acid catalysis. MSNs with high specific surface area, pore volume, and hydrothermal stability have been commonly considered in research^6^. On the other hand, adsorption is considered one of the promising desulfurization methods for removing organic sulfur compounds in petroleum distillates, as it is relatively cheap and can be applied at atmospheric pressure and ambient temperature^7^. Also, selective recognition and adsorption of target pollutants from complex matrices before the detection of trace amounts of them is frequently required^8^. Accordingly, molecularly imprinted polymers (MIPs) show great potential in adsorptive desulfurization^9,10^. Molecular imprinting is a powerful synthesis method for forming selective recognition sites in MIP through the self-assembly of the functional monomers and template molecule/s via non-covalent interactions or reversible covalent binding of the functional groups of the templates and those of the complementary monomers. Subsequently, the resulting complexes are copolymerized with an appropriate cross-linking agent. After the removal of the template from the cross-linked polymer matrix, many cavities complementary to the size and shape of the template are formed^11^. The conventional techniques used in the preparation of MIPs, such as precipitation polymerization (PP), suspension polymerization (SP), and bulk polymerization (BP)^12^, usually have some drawbacks, such as low adsorption capacity, poor site accessibility, inefficient mass transfer, and inappropriate rebinding ability. To overcome these disadvantages, some new technologies related to surface molecular printing (SMP) have been recently developed^13–17^. Morshedy et al.^18^ synthesized a novel surface-imprinted polymer (MIP/AgPx) with different amounts of silver phosphate and used it in photocatalytic desulfurization of real diesel fuel. Qin et al.^19^ prepared a SMIP grafted on mesoporous carbon nanospheres (SMIP/OMCNS) for deep desulfurization of fuel. Wang et al.^20^ synthesized a double template MIP on the surface of magnetic silica (Fe_3_O_4_@SiO_2_@DT-MIP) for the removal of dibenzothiohphene (DBT) and 4-methyldibenzothiophene (4-MDBT) from gasoline. The surface modification of MSNs with organic functional groups is the first step towards synthesizing silica-polymer nanocomposites^21–23^. One common method to modify and functionalize the surface of silica nanoparticles is the reaction of an organosilane with surface silanol groups. The organosilane coupling agent chemically attaches amine groups (–NH_2_) to silicon-based materials. These grafted organosilanes allow for obtaining modified silica for monomer polymerization^22^. Hu et al.^24^ chemically modified the surface of silica gel with methacrylic acid (MAA) and γ-aminopropyltriethoxysilane and prepared a MIP for the selective recognition of benzothiophene. The synthesis of suitable MIP nanocomposites with combined oxidation and adsorption properties has significant effect on increasing the efficiency of desulfurization compared to a single oxidation or adsorption process. In the present work, a new and efficient organic–inorganic hybrid nanocomposite was designed with combined oxidation and adsorption properties based on surface molecularly imprinted polymer and MSNs using CCD. The CCD experiments were performed to obtain the maximum desulfurization percentage by applying three parameters: the weight percentage of oxidative catalyst (HPW, wt% X_1_) incorporated in MCM-48, the template amount required for synthesis of surface imprinted polymer (DBT, mmol, X_2_) and the oxidant/sulfur molar ratio (X_3_) used during the desulfurization process. First, MCM-48 supported HPW was prepared at different HPW weight percentages. On the surface of MCM-48-HPW modified with APTMS, a molecularly imprinted polymethacrylic aid for the selective adsorption of DBT was formed by using MAA as the functional monomer, EGDMA as the crosslinker, and different amounts of DBT as the template molecule. Finally, various oxidant/sulfur molar ratios were applied during the desulfurization reaction to optimize desulfurization efficiency according to CCD.

## Results and discussions

### Central composite design (CCD) approach for optimization of oxidative/adsorptive desulfurization results

The CCD design was performed using Design Expert Software 11 to evaluate the effect of the parameters A (X_1_): Catalyst percentage (HPW, wt. %), B (X_2_): Template amount (DBT, mmol), and C (X_3_): Oxidant/sulfur molar ratio, on the percentage desulfurization (Y%) of model fuels using polymer nanocomposites to obtain maximum desulfurization efficiency. Polymer nanocomposites were synthesized by varying the HPW weight percentage (20–40 wt%) and template amount (0.05–0.1 mmol) according to the CCD. Using oxidant/sulfur molar ratio as the third parameter (between 4 and 10), a total of 20 experiments (five levels for each parameter) were performed using the equation ${2}^{k}+2k+6$, including 6 axial points, 8 factorial points, and 6 replicates at the center point, where $k$ is the number of parameters. The axial points were placed at (± α, 0, 0), (0, ± α, 0) and (0, 0, ± α) from the center point, and the value of α was 1.682 to allow for rotatability in the design space. Table 1 shows the levels of the independent variables.

**Table 1:** Independent variables and their levels.

Independent variables	Symbols	Levels				
		(− α)	(− 1)	0	(+ 1)	(+ α)
Catalyst percentage (HPW, wt. %)	A	13.1812	20	30	40	46.8179
Template amount (DBT, mmol)	B	0.0329	0.05	0.075	0.1	0.1170
Oxidant/Sulfur molar ratio	C	1.9546	4	7	10	12.0454

**Figure 1:**
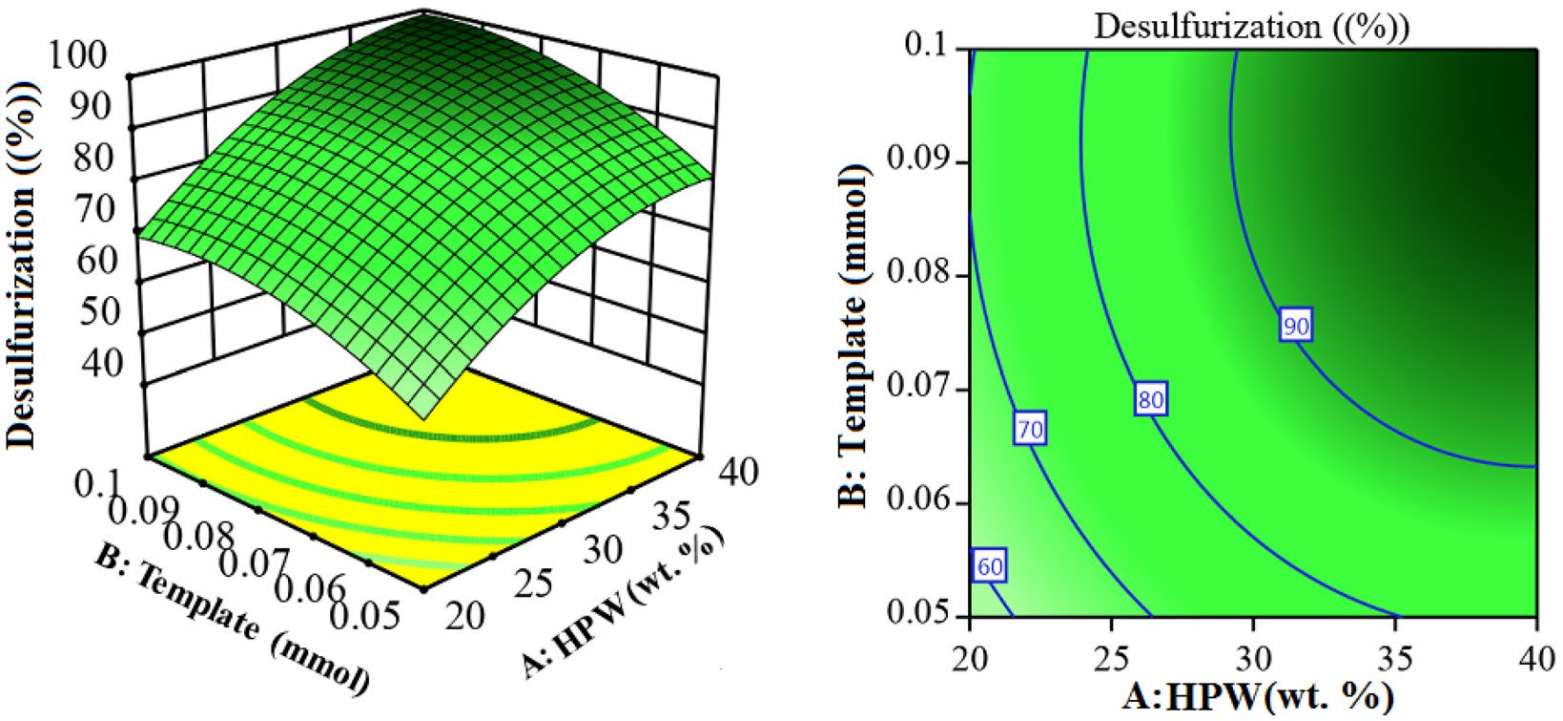
Surface (left) and contour (right) plots for the effects of HPW wt. % and template amount on the desulfurization percentage.

The design matrices included twenty desulfurization experiments, and the corresponding responses are shown in Supplementary Table S1. The experimental data were inserted into the software and fitted to linear, 2F, quadratic, and cubic regression models. According to the insignificant lack of fit (Supplementary Table S2) and model summary statistics results (Supplementary Table S3), the quadratic polynomial model (Eq. 1) was suggested as the best model to correlate independent variables to the dependent variable:

1 $$\mathrm{Y}\left(\%\right)={b}_{0}+{b}_{1}{x}_{1}+{b}_{2}{x}_{2}+{b}_{3}{x}_{3}+{b}_{11}{x}_{1}^{2}+{b}_{22}{x}_{2}^{2}+{b}_{33}{x}_{3}^{2}{+b}_{12}{x}_{1}{x}_{2}+{b}_{13}{x}_{1}{x}_{3}+{b}_{23}{x}_{2}{x}_{3}$$

here ${x}_{1}$, ${x}_{2}$, and ${x}_{3}$ are the independent variables, Y is the response, and ${\mathrm{b}}_{0}$ is the intercept coefficient. ${\mathrm{b}}_{1}$, ${\mathrm{b}}_{2}$ and ${\mathrm{b}}_{3}$, ${b}_{11}$, ${b}_{22}$ and ${b}_{33}$ and ${b}_{12}$, ${b}_{13}$ and ${b}_{23}$ give the coefficients of squared, linear, and interaction effects terms, respectively. The experimental data were tested entirely with analysis of variance (ANOVA) to evaluate the adequacy of the model and to estimate the effects of the main variables and their interaction on the response, and the results are shown in Supplementary Table S4. The *p*-value evaluates the significance of each model. A model is considered significant if the *p*-value is < 0.05 (95% confidence level). From the ANOVA results, a very low *p*-value (< 0.0001) and a high F-value (268.62) imply that the model is extremely significant for oxidative/adsorptive desulfurization. For percentage desulfurization, A, B, C, AB, AC, BC, A^2^, B^2^, and C^2^ are significant model terms. Additionally, parameters A and B were highly statistically significant, as they had very low *p*-values and very high F-values. The desulfurization percentage predicted by the model versus the experimental desulfurization was plotted on the graph and shown in Supplementary Fig. S1. It can be seen that there is a good agreement between the predicted and the experimental data. The quadratic model obtained for the percentage desulfurization using polymer nanocomposites in terms of coded values is presented in Eq. (2).

2 $$ \begin{gathered} {\mathbf{Y}}\left( {{\text{Desulfurization}}\;\% } \right) = + {88}.{33} + {13}.{62}\;{\text{A}} + {7}.{81}\;{\text{B}} + {1}.{59}\;{\text{C}} + {1}.{12}\;{\text{AB}} + {2}.{92}\;{\text{AC}}{-}{1}.{75}\;{\text{BC}} \hfill \\ \quad {-}{6}.{6}0\;{\text{A}}^{{2}} {-}{5}.{27}\;{\text{B}}^{{2}} {-}{4}.{23}\;{\text{C}}^{{2}} \hfill \\ \end{gathered} $$

where A is catalyst percentage, B is template amount, and C is oxidant/sulfur molar ratio. In interaction terms (AB, AC, and BC), a positive sign indicates that two parameters have a synergistic effect, while a negative sign shows that one parameter positively and another parameter negatively affects the response. As given by Eq. (2), the coefficient of A was higher than that of B and C, indicating that the effect of parameter A was greater than that of B and C. After constructing the model, surface and contour plots were obtained to visualize the combined effects of independent variables on the response. Supplementary Figs. S2 and S3 show the response surface plot (left) and contour plot (right) of desulfurization efficiency as a function of A-B, A-C, and B-C, respectively. At low levels of both HPW weight percentage and template amount, the surface plot (Fig. 1, left) displays a minimum oxidative/adsorptive desulfurization (< 60%), and this can be associated with insufficient active oxidation sites in the catalyst and a limited number of binding sites in the imprinted polymer. Increasing the HPW weight percentage and maintaining a constant template concentration significantly enhances the desulfurization efficiency above 80%. The desulfurization percentage was increased from < 60% to around 70% by increasing the template amount from 0.05 to 0.1 mmol at 20 wt% HPW. This showed that the surface-imprinted polymer with selective cavities improves the recognition and adsorption of target DBT molecules and has a positive effect on desulfurization efficiency, which is lower than the HPW percentage. The positive sign before the AB interaction term in Eq. (2) confirms a synergistic effect of HPW wt% and template amount on the response. Accordingly, imprinted cavities make active oxidation sites of MCM-48-HPW available to DBT molecules, resulting in an increased efficiency of the oxidation catalyst. This synergistic effect provides a new way to effectively remove the DBT from fuels. Supplementary Fig. S2 represents the combined effect of HPW wt. % and oxidant/sulfur molar ratio on desulfurization percentage. It was observed that at low weight percentages of HPW, increasing the oxidant/sulfur molar ratio from 4 to about 7 gradually increased the desulfurization percentage.

However, desulfurization efficiency remains constant at molar ratios higher than seven. This phenomenon could be related to the fact that the initial water in the H_2_O_2_ (30 wt% H_2_O_2_), the water created from the oxidative desulfurization reaction, and the water produced from the thermal decomposition of the oxidant can be adsorbed on active sites of HPW immobilized on MCM-48, thus reduce desulfurization performance. However, at high weight percentages of HPW, desulfurization efficiency increased significantly with increasing oxidant/sulfur molar ratio. HPW weight percentage and oxidant/sulfur molar ratio showed a significant synergistic effect on desulfurization efficiency, according to the large positive coefficient for the AC interaction term (Eq. (2)). Supplementary Fig. S3 shows the combined effect of template amount and oxidant/sulfur molar ratio on desulfurization percentage. At oxidant/sulfur molar ratios less than 7, the desulfurization percentage moderately increased with increasing template amount. Whereas, at oxidant/sulfur molar ratios greater than 7, the desulfurization percentage first increased from 73 to 87 and then slightly decreased, according to the negative sign of the BC interaction term.

### Determination and validation of optimum conditions

The optimization function of Design Expert 11 software was used to predict the optimum conditions for desulfurization percentage. Supplementary Table S5 gives the lower and upper limits of variables, their importance, and goals used to obtain the response. The range definition for the parameters was based on the primary results. Of the 97 proposed solutions, the first three solutions with a desirability function of 1 were chosen to confirm the model accuracy using the obtained empirical model Eq. (2). Supplementary Fig. S4 displays the first solution proposed by the model for predicting the optimum desulfurization conditions using the numerical feature and the results of verification experiments conducted at optimum conditions are shown in Table 2. The obtained results confirmed the high predictability of the model for desulfurization in experimental conditions. The resulting nanocomposite at optimum conditions was denoted as SMIP-PMAA@MCM-48-HPW and was characterized by different analysis methods. In addition to optimal nanocomposite, the MCM-48-xHPW (x = 39.99 wt%), bulk MIP-PMAA and non-imprinted polymer nanocomposite (NIP-PMAA/MCM-48-HPW) were prepared and their desulfurization ability was investigated. The NIP-PMAA@MCM-48-HPW was synthesized in an order similar to the SMIP-PMAA@MCM-48-HPW, except with the addition of the template. The MCM-48-xHPW synthesized using 39.99 wt% HPW was denoted as MCM-48-HPW and the amine-functionalized MCM-48-HPW was denoted as NH_2_-MCM-48-HPW.

**Table 2:** The first three solutions at optimum conditions.

Experiments	Optimal levels of independent variables	Optimized values	Experimental values
		Y%	Y%
1	A(wt%) = 39.998, B(mmol) = 0.096, C = 6.941	99.071	98.540
2	A(wt%) = 39.032, B(mmol) = 0.097, C = 8.723	99.055	98.021
3	A(wt. %) = 39.431, B(mmol) = 0.089, C = 8.050	99.224	98.418

### Characterization of optimized polymer nanocomposite

#### Crystal structure and morphology characterization

**Figure 2:**
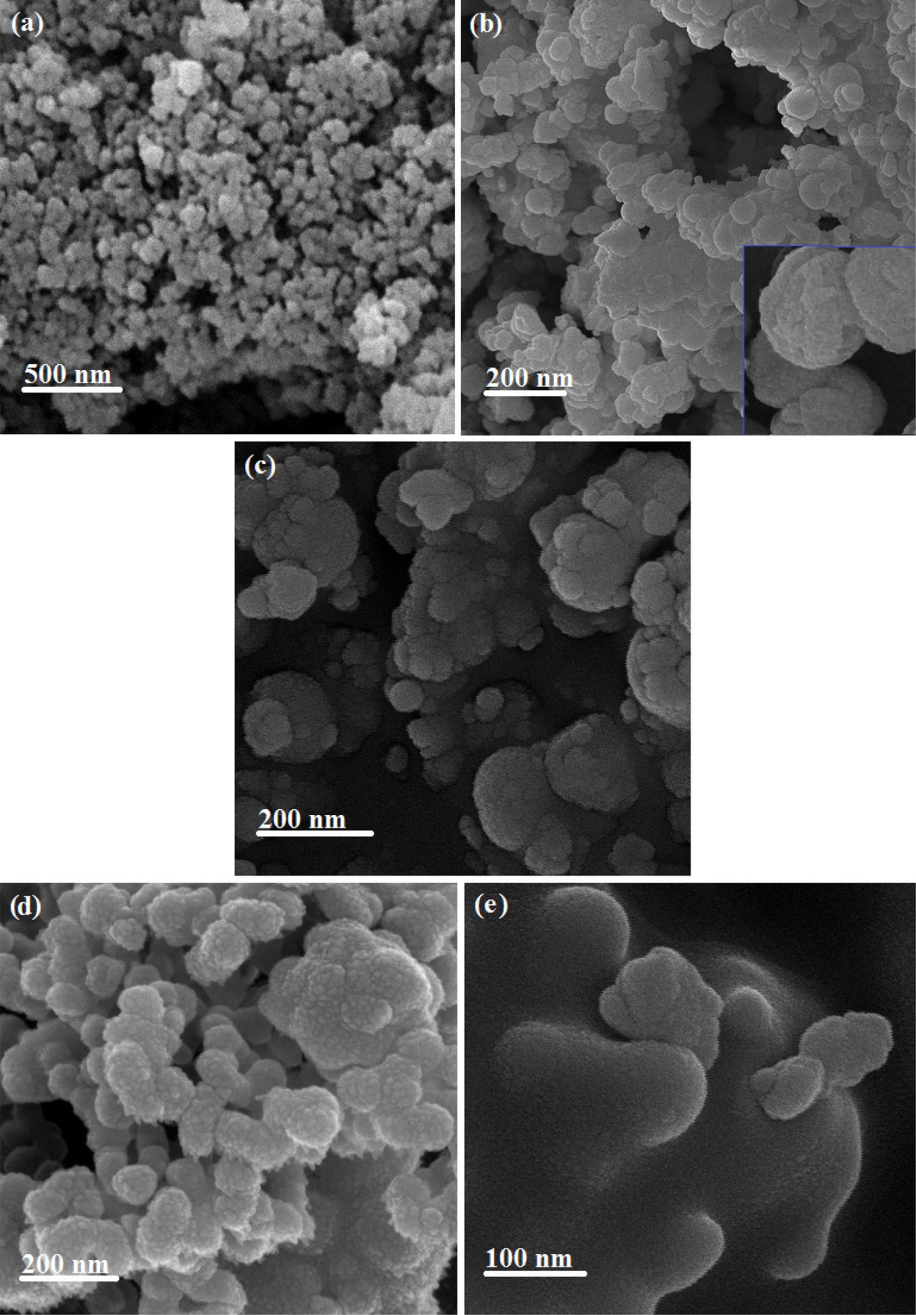
SEM images of MCM-48 (**a**), MCM-48-HPW (**b**), NH_2_-MCM-48-HPW (**c**) SMIP-PMAA@MCM-48-HPW (**d**) and NIP-PMAA@MCM-48-HPW (**e**).

The structure of prepared samples was confirmed with low-angle XRD analysis and shown in Supplementary Fig. S5. The XRD pattern of MCM-48-HPW clearly shows the characteristic diffraction peaks of MCM-48 type of mesoporous silica nanoparticles, as described in previous work^25,26^. The absence of diffraction peaks of the crystalline HPW phase in the XRD pattern of the MCM-48-HPW suggests that HPW is well dispersed within the MCM-48. In NH_2_-MCM-48-HPW, the intensity of the peaks was markedly decreased due to the grafting of APTMS to MCM-48-HPW, confirming the loose of a partial space related to the pores by the introduction of the NH_2_ groups. However, the original crystal structure was maintained. In the XRD pattern of SMIP-PMAA@MCM-48-HPW, the main characteristic peak of the MCM-48 (211) is still preserved in the polymer nanocomposite. However, as the polymer layer was formed on the surface of the NH_2_-MCM-48-HPW, the peak intensity was significantly reduced. This phenomenon may be attributed to the formation of organic–inorganic network structures^27^. Figure 2 exhibits the SEM images of pure mesoporous MCM-48 (a), MCM-48-HPW (b), NH_2_-MCM-48-HPW (c), the optimum SMIP-PMAA@MCM-48-HPW nanocomposite after elution (d) and NIP-PMAA@MCM-48-HPW (e). It is seen that mesoporous MCM-48 has a uniform spherical shape with an average diameter of less than 100 nm with a rough surface inclined to agglomeration (Fig. 2a). MCM-48-HPW also displayed a sphere-like morphology (Fig. 2b), while the particle size of NH_2_-MCM-48-HPW was found to be bigger and non-uniform upon modification (Fig. 2c). The increase in particle size is attributed to APTMS grafting on the surface of MCM-48-HPW, resulting in the growth of the particles^28^. However, particles show a tendency to aggregate among themselves, probably due to the silica dispersion in toluene, where they are not soluble. The surface of optimum SMIP-PMAA@MCM-48-HPW nanocomposite exhibits a rough morphology, and abundant pores are seen on the surface. The spherical shapes are silica nanoparticles, which are decorated by polymer. Elution and extraction of DBT molecules from the surface provide a rough surface with imprinted cavities (Fig. 2d). The SEM image of the non-imprinted polymer nanocomposite (NIP-PMAA/MCM-48-HPW) reveals an uniform, non-porous and smooth structure all over the surface, according to the previous work^29^, confirming the successful preparation of the non-imprinted polymer nanocomposite (Fig. 2e).

**Figure 3:**
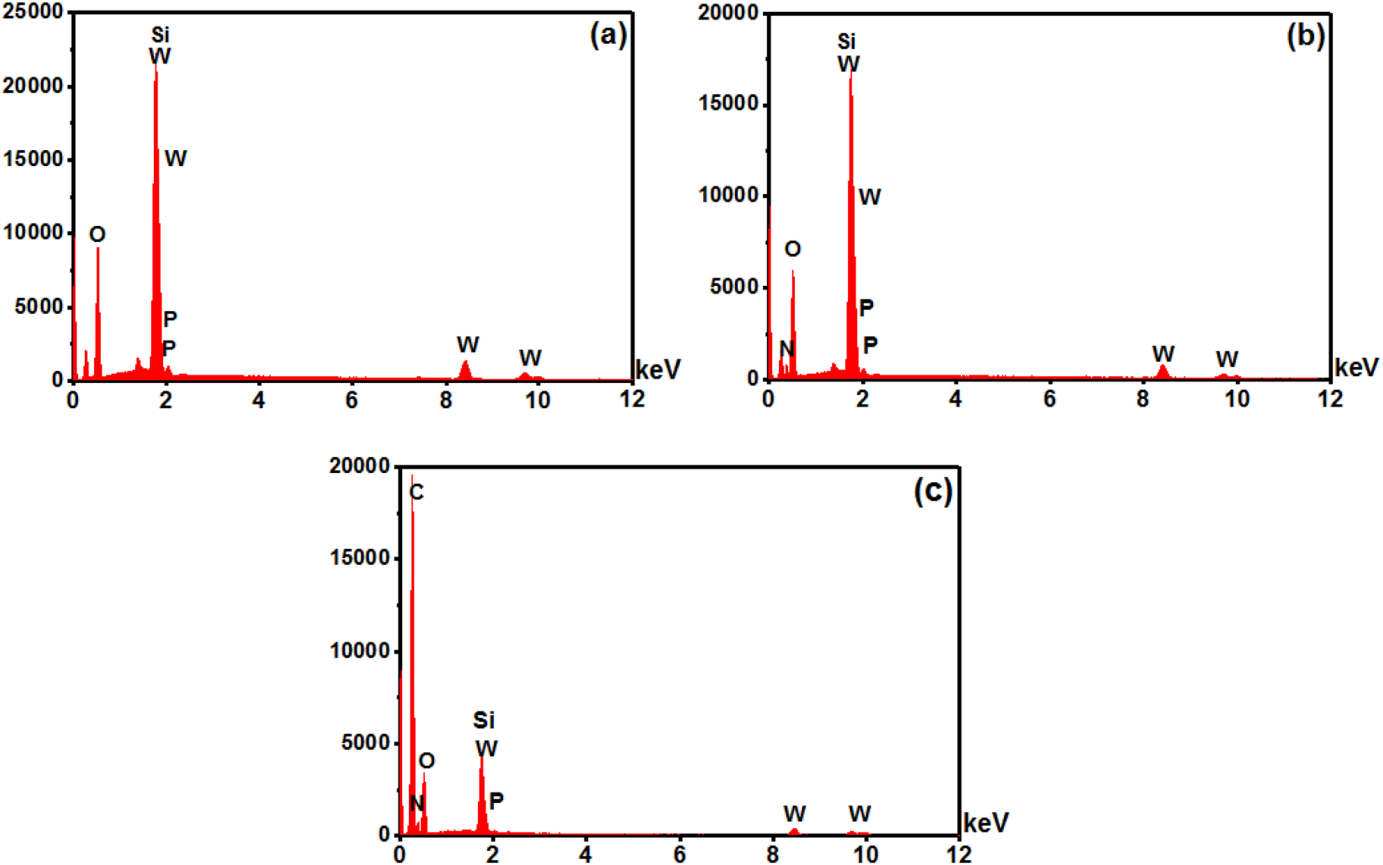
EDX analysis of (**a**) MCM-48-HPW, (**b**) NH_2_-MCM-48-HPW and (**c**) SMIP-PMAA@MCM-48-HPW.

The elemental composition of MCM-48-HPW, NH_2_-MCM-48-HPW, and SMIP-PMAA@MCM-48-HPW samples obtained by energy dispersive X-ray analysis (EDX) is shown in Fig. 3. The EDX spectrum of MCM-48-HPW clearly shows the expected main elements (O, Si) of SiO_2_ nanoparticles. The presence of W and P in the MCM-48-HPW was also confirmed (Fig. 3a). There was a small characteristic peak for the nitrogen element of amine from EDX analysis of NH_2_-MCM-48-HPW compared to MCM-48-HPW, confirming the presence of amine groups on the surface of amine-modified silica nanoparticles (Fig. 3b). The strong signal related to the C atom at the spectrum of SMIP-PMAA@MCM-48-HPW confirms that the polymer is successfully grafted on the amine-functionalized MSNs surface (Fig. 3c).

**Figure 4:**
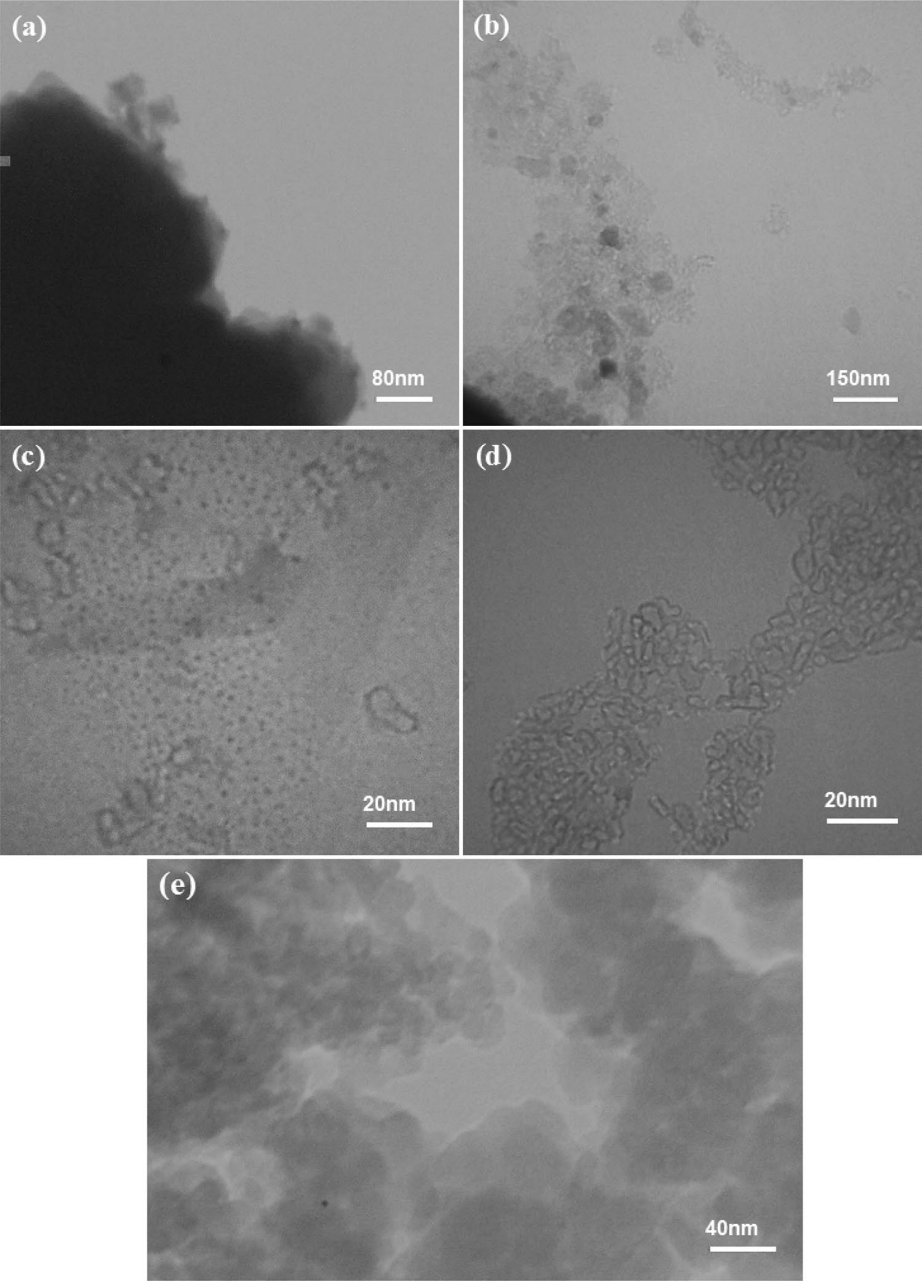
TEM images of NH_2_-MCM-48-HPW (**a**), SMIP-PMAA@MCM-48-HPW before removal of DBT (**b**), the magnified TEM image of the imprinted polymer layer coated on the surface of silica nanoparticles in SMIP-PMAA@MCM-48-HPW before DBT elution (**c**), after DBT elution (**d**) and the magnified TEM image of non-imprinted polymer coated on the surface of silica nanoparticles in NIP-PMAA@MCM-48-HPW (**e**).

The detailed morphology of prepared samples was studied using TEM analysis. TEM images of NH_2_-MCM-48-HPW and SMIP-PMAA@MCM-48-HPW before the removal of DBT are shown in Fig. 4a and b, respectively. Magnified TEM images of the polymer layer coated on the surface of silica nanoparticles in SMIP-PMAA@MCM-48-HPW before and after the removal of DBT are shown in Fig. 4c and d, respectively. Compared to NH_2_-MCM-48-HPW, the TEM image of SMIP-PMAA@MCM-48-HPW clearly showed that a polymer layer was coated on silica nanoparticles, confirming the successful synthesis of the surface MIP polymer nanocomposite. The loaded imprinted polymer (Fig. 4c) was observed to have dark dots, which can be related to the filling of holes by DBT during the polymerization. The magnified image of the non-imprinted polymer coated on the surface of silica nanoparticles in NIP-PMAA@MCM-48-HPW (Fig. 4e) shows a relatively dense, uniform, and non-porous structure compared to SMIP-PMAA@MCM-48-HPW (Fig. 4b), which can be attributed to the absence of imprinted cavities.

#### FTIR analysis

**Figure 5:**
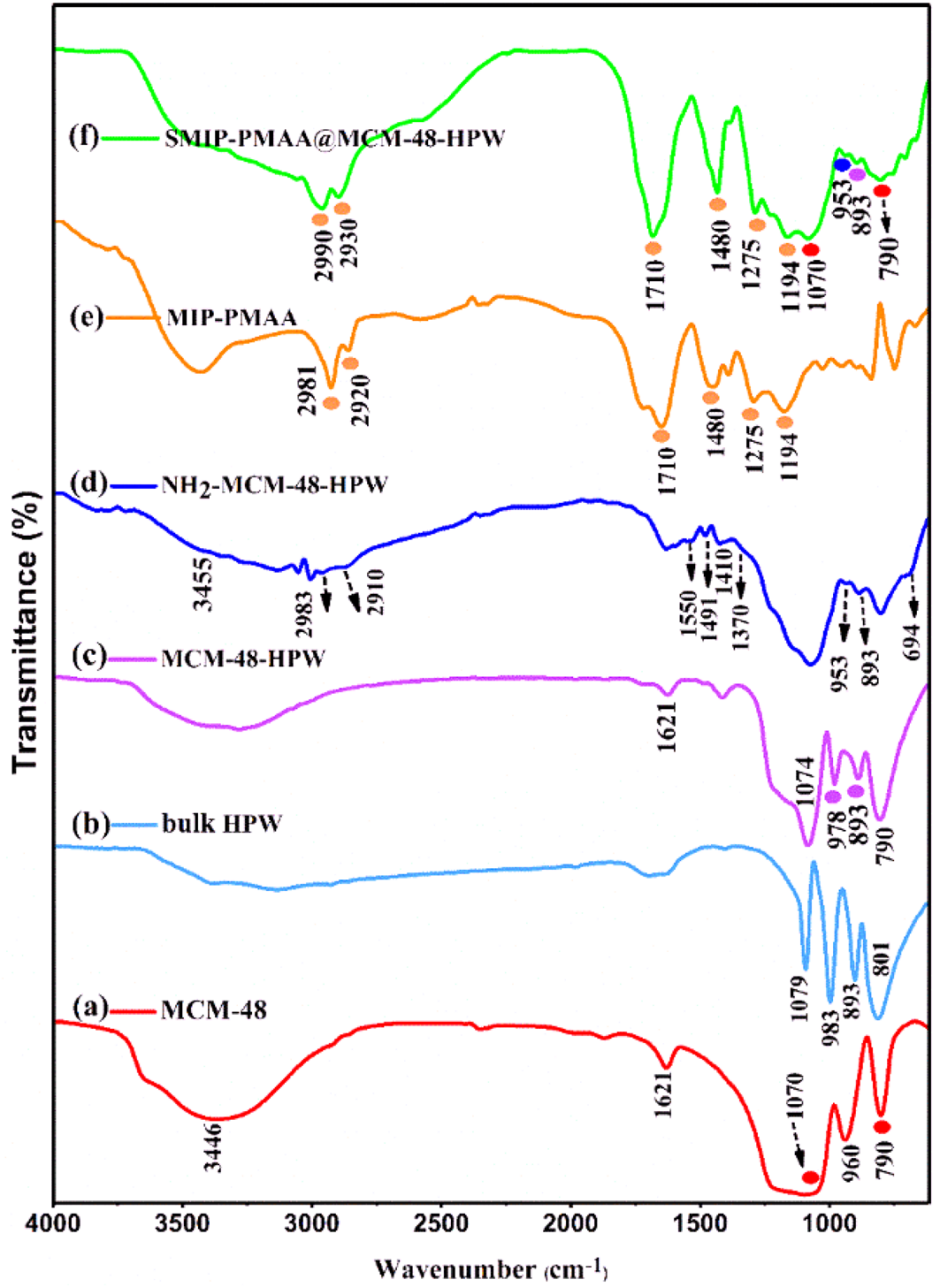
FT-IR spectra for a pure MCM-48 (**a**), bulk HPW (**b**), MCM-48-HPW (**c**), NH_2_-MCM-48-HPW (**d**), bulk MIP-PMAA (**e**) and SMIP-PMAA@MCM-48-HPW (**f**).

The detailed results of FT-IR characterization are shown in Fig. 5. In the pure MCM-48 (Fig. 5, a), the broad bond around 1070 cm^−1^ and the vibration band at 790 cm^−1^ are assigned to the asymmetric and symmetric stretching of Si–O–Si, respectively. The weak peak at 960 cm^−1^ is assigned to the symmetric stretching vibration of Si–OH bands^30^. In the case of bulk HPW (Fig. 5, b), four characteristic bands at 1079, 983, 893, and 801 cm^−1^ are assigned to the stretching vibrations of P–O, W = O_terminal_, W–O_corner_–W, and W–O_edge_–W, respectively^26,31,32^.

In the MCM-48-HPW sample (Fig. 5, c), the intensity of HPW characteristic peaks was weaker than that of the bulk HPW. Also, the bands of 1079 and 983 cm^−1^ were shifted to 1074 and 978 cm^−1^, respectively, which could be explained by interference from the characteristic bands of mesoporous silica (1070 and 960 cm^−1^). However, the band of vibration of W–O_corner_–W at 893 cm^−1^ appeared clearly after incorporating it into MCM-48^33^. In the NH_2_-MCM-48-HPW sample (Fig. 5, d), the stretching vibrations between 2850 and 3050 cm^−1^ and bending vibrations at 1410–1491 cm^−1^ are related to the –C–H groups from APTMS and the N–H bending vibration band around 1550 cm^−1^ implies the successful grafting of amine groups onto the MSNs surface^24^. The shift of the peak at 978 to 953 cm^−1^ shows an interaction between silanol groups of MSNs and APTMS^34,35^. The spectrum of bulk MIP-PMAA polymer (after template elution) is also shown in Fig. 5, e. The bands between 2990 and 2900 cm^−1^ are assigned to C–H asymmetric stretching of –CH_2_– and –CH_3_– groups in the polymer chain and cross-linking reagent (EGDMA). The bands at 1194 cm^−1^ and 1275 are attributed to the stretching of O–C(O)–C from EGDMA and sp^2^ C–O from segments of MAA, respectively^36,37^. As shown in Fig. 5, f, in the SMIP-PMAA@MCM-48-HPW, the peaks associated with the structure of the silica nanoparticles (790 and 1070 cm^−1^) were preserved. However, their intensities were reduced due to surface polymerization. Compared to NH_2_-MCM-48-HPW, the SMIP-PMAA@MCM-48-HPW exhibited apparent CH_2_ and CH_3_ stretching vibrations between 2900 and 2990 cm^−1^. These stretching modes could also be found in the MIP-PMAA spectra. The stretching vibrations of C=O (1710 cm^−1^), –CH_2_ (1480 cm^−1^), C–O (1275 cm^−1^), and C–C(O)–C (1194 cm^−1^) are consistent with those of MIP-PMMA also revealed in SMIP-PMAA@MCM-48-HPW. The above spectrum changes sufficiently show that the monomers of MAA have been grafted and polymerized onto the surfaces of silica nanoparticles. Based on the obtained results, it can be concluded that the fabrication procedure of the polymer nanocomposite has been successfully performed.

#### Textural properties analysis

The textural properties of the prepared samples are presented in Supplementary Fig. S6 and Table 3. The BET surface area, total pore volume and BJH pore size distribution of the pure MCM-48 with type IV isotherm were 279 m^2^g^−1^, 0.3124 m^3^g^−1^ and 6.23 nm, respectively. After introducing HPW, MCM-48-HPW retained its mesoporous structure (the isotherm was type IV). However, decreased surface area (238 m^2^g^−1^), total pore volume (0.2910 cm^3^g^−1^) and pore size (5.06 nm) relative to the pure MCM-48 are attributed to the deposition of HPW within the mesopores of the MCM-48 support. NH_2_-MCM-48-HPW, SMIP-PMAA@MCM-48-HPW and NIP-PMAA/MCM-48-HPW also showed a type IV isotherm, indicating the characteristic of mesoporous solids^38^.

**Table 3:** Textural properties of synthesized materials.

Sample	S_BET_ (m^2^g^−1^)	V_Total_ (cm^3^g^−1^)	V_micro_ (cm^3^g^−1^)	V_meso_ (cm^3^g^−1^)	Average pore diameter (nm)
(1) MCM-48	279	0.3124	0.0519	0.2605	6.23
(2) MCM-48/HPW	238	0.2910	0.0455	0.2455	5.06
(3) NH_2_-MCM-48-HPW	156	0.2058	0.0315	0.1743	3.92
(5) SMIP-PMAA@MCM-48-HPW	295	0.3417	0.0301	0.3116	8.24
(4) NIP-PMAA@MCM-48-HPW	209	0.2635	0.0384	0.2251	4.53

A significant reduction in surface area, pore diameter and total pore volume of NH_2_-MCM-48-HPW (S_BET_ = 156 m^2^g^−1^, V_Total_ = 0.2058 cm^3^g^−^1 and pore size = 3.92 nm) relative to MCM-48-HPW confirms that amine functionalization partly occurred inside the pores and tunnels in silica nanoparticles were covered. The MIP nanocomposite showed a higher surface area (295 m^2^g^−1^), total pore volume (0.3417 cm^3^g^−1^) and pore size (8.24 nm) than the NIP nanocomposite (S_BET_ = 209 m^2^g^−1^, V_Total_ = 0.2635 cm^3^g^−1^, average pore diameter = 4.53 nm) because numerous cavities of the surface-imprinted polymer enriched the porous structure of SMIP-PMAA@MCM-48-HPW. The total results indicated that the imprinted holes were successfully formed on the silica surface in the SMIP-PMAA@MCM-48-HPW sample. In the ^1^H NMR spectrum of SMIP-PMAA@MCM-48-HPW shown in Supplementary Fig. S7, peaks at δ = 0.85–1.42 and δ = 1.8–2.3 ppm are attributed to the protons of methyl (–CH_3_–) and methylene (–CH_2_–) groups of poly MAA^39,40^, respectively, indicating the formation of the polymer on the surface of the MCM-48-HPW. The weak peaks appearing at 1.52 (–C–CH_2_–C–) and 2.41 (–N–CH_2_–C–) ppm can be assigned to the protons of the propyl chain of APTMS grafted on the surface of MCM-48-HPW silica^41^.

#### Thermogravimetric analysis (TGA)

**Figure 6:**
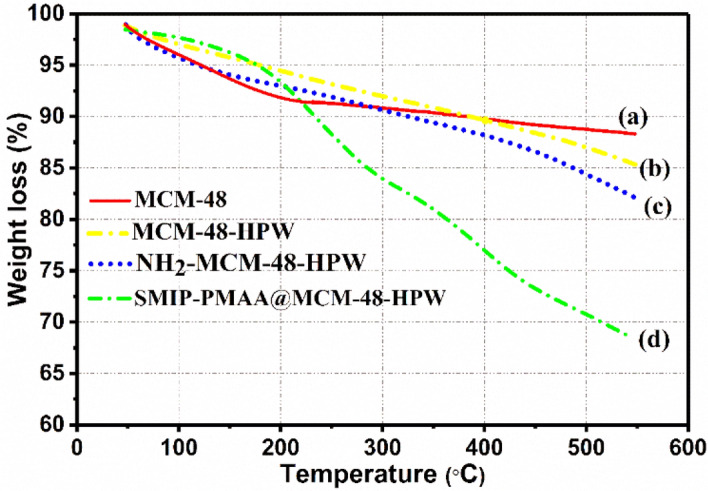
TGA curves of (**a**) MCM-48, (**b**) MCM-48-HPW, (**c**) NH_2_-MCM-48-HPW, and (**d**) SMIP-PMAA@MCM-48-HPW.

The presence of aminopropyl groups and coated polymers on the silica surface can be estimated by TG. As shown in Fig. 6, the observed weight loss of 6 wt% up to 200 °C in MCM-48 (Fig. 6a) is due to the removal of physically adsorbed water. A further weight loss (4.1 wt%) between 200 and 550 °C is probably due to the decomposition of the template, which is not entirely removed and the dehydroxylation of the silanol group in MCM-48 nanoparticles. In the case of MCM-48-HPW (Fig. 6b), the weight loss below 200 °C (3.5 wt%) is mainly caused by the non-coordinated H_2_O molecules of HPW. Compared with MCM-48, a higher mass loss of about 9 wt% for MCM-48-HPW between 200 and 550 °C can be explained by the removal of crystalline H_2_O molecules present in HPW inside the channels of MCM-48, the beginning of breakdown and decomposition of H_3_PW_12_O_40_ structure and the loss of relatively stable acidic protons.

The TG curve of NH_2_-MCM-48-HPW (Fig. 6c) gave a total mass loss of about 10.6 wt. % between 200 and 550 °C, which could be attributed to the simultaneous decomposition of the kegging structure and aminosilanes grafted onto MCM-48-HPW surfaces. The TGA curve for the SMIP-PMAA@MCM-48-HPW nanocomposite revealed three weight loss regions (Fig. 6d). The first region at temperatures below 200 °C was due to the loss of physisorbed water. The second region between 200 and 300 °C accounted for the stripping of carboxylic acid and hydroxyl groups in the branched chain of PMAA polymers. The third region around 450 °C can be attributed to the chain scission of the PMMA macromolecules and the cleavage of the backbone polymer. The TG curve of SMIP-PMAA@MCM-48-HPW gave a total mass loss of about 24.7 wt% between 200 and 550 °C.

#### Process of oxidative/adsorptive desulfurization by polymer nanocomposites

To study the efficiency of oxidation/adsorptive desulfurization, the sulfur compound (DBT) in the model fuel before and after reactions was measured by HPLC. After the desulfurization reaction, the upper phase of the model fuel was withdrawn and injected into the HPLC. Supplementary Fig. S8 shows the HPLC analysis of DBT in the model fuel before, during, and after desulfurization using SMIP-PMAA@MCM-48-HPW. By comparing the DBT peaks in the chromatograms, it can be seen that the DBT peak disappeared largely after desulfurization, indicating nearly complete removal of DBT from the model fuel. Moreover, no oxidized sulfur species were found in the model fuel due to the complete extraction of the corresponding sulfone into the aqueous phase (acetonitrile). The desulfurization experiments were also conducted using the bulk HPW, MCM-48-HPW, bulk MIP-PMAA and NIP-PMAA@MCM-48-HPW at conditions reported in Table 4. The comparison of desulfurization efficiencies in Table 4 shows that the SMIP-PMAA@MCM-48-HPW presents a higher desulfurization yield (98.54%) than the NIP nanocomposite (72.19%) under the same experimental conditions (except for the addition of the template), strongly indicating an imprinting effect. The enhanced desulfurization performance of the optimum MIP nanocomposite can be attributed to its simultaneous oxidation and adsorption abilities. Having a large surface area, the optimum SMIP-PMAA@MCM-48-HPW nanocomposite can facilitate access to active sites and show improved adsorptive capacity compared to the single catalyst or adsorbent.

**Table 4:** Comparison of desulfurization efficiency.

Sample	Catalyst (HPW, wt. %)	Template amount (mmol)	H_2_O_2_/Sulfur ratio	Desulfurization (%)
HPW (bulk)	100.00	–	6.94	41.50
MCM-48-HPW	39.99	–	6.94	69.35
MIP-PMAA (bulk)	–	0.096	–	38.71
SMIP-PMAA@MCM-48-HPW	39.99	0.096	6.94	98.54
NIP-PMAA@MCM-48-HPW	39.99	–	6.94	72.19

#### Mass balance experiments

In this study, imprinted polymer nanocomposites were prepared with specially designed recognition cavities that can interact with DBT molecules through hydrogen bonds, shape selection, and electrostatic interactions. The chemisorbed DBT molecules (through hydrogen bonding) can be oxidized to their corresponding sulfone during the ODS reaction and extracted into the acetonitrile phase. The identification of DBT sulfone in acetonitrile phase is a reliable indicator of the oxidation of DBT molecules. On the other hand, some DBT molecules can be recognized and captured by the cavities of the molecularly imprinted polymer. Additionally, a small amount of DBT molecules can be extracted into acetonitrile phase and removed. Therefore, the total DBT removal can be a combination of trapping some DBT molecules in the imprinted cavities, oxidizing some DBT molecules that are chemisorbed on the surface sites, and extracting some DBT into acetonitrile phase. To evaluate the sulfur mass balance, DBT was determined in all phases of the desulfurization process, including the model fuel, the aqueous phase (methanol/acetic acid), and the extraction solvent. The amounts of DBT and its oxidation product (DBT sulfone) extracted into the acetonitrile phase were determined using the HPLC analysis method, applying the calibration method (Supplementary Fig. S9). Also, after the desulfurization experiment, the optimum nanocomposite was separated by filtrating and washed with methanol/acetic acid (9:1) to extract DBT. The eluent was analyzed by a sulfur analysis instrument to determine the DBT trapped in the imprinted polymer. Finally, the residual concentration of the DBT in the organic phase was determined using HPLC. Supplementary Fig. S10 and Table S6 show the schematic figure of the DBT mass balance and the mass balance results for 1000 ppm DBT in the desulfurization process, respectively. As can be seen, using the optimum SMIP-PMAA@MCM-48-HPW, the extraction solvent contained about 64.45% of DBT, methanol/acetic acid contained about 30.48% and only 1.46% remained in the model fuel.

#### Desulfurization of real fuel oil and recycling of the polymer nanocomposite

The oxidative/adsorptive desulfurization of a real fuel sample (gasoline) was also investigated using the optimum nanocomposite under optimized conditions. The total sulfur content in the real fuel was 1240 mg/L. The amounts of DBT before and after the reaction were analyzed by a Gas Chromatograph–Mass Spectrometer (GC–MS). The DBT peak (m/z = 184) was detected for the gasoline sample before desulfurization (see Supplementary Fig. S11a). The results in Supplementary Fig. S11b show that after desulfurization, a small peak for DBT is detected. The findings declared that 96% removal efficiency was achieved for the real fuel. The reusability investigation of the optimum nanocomposite was also performed for the gasoline samples. After each desulfurization process, the SMIP-PMAA@MCM-48-HPW was separated by filtration, washed with toluene and methanol/acetic acid (9:1) and dried at 50 °C for 10 h. The SEM image of the spent optimum imprinted polymer nanocomposite confirms its stable structure and morphology after five times recycling (Supplementary Fig. S12a). The FTIR spectrum of the spent polymer nanocomposite before DBT elution confirms that DBT molecules have been successfully combined with the functional monomers (Supplementary Fig. S12b). As can be seen, a strong absorption band is observed at 730 cm^−1^ which can be attributed to the out-of-plane bending vibration of –CH on benzene rings in the un-eluted imprinted polymer nanocomposite. Additionally, a vibration band at 1406 cm^−1^ is related to the thiophene ring vibration^42,43^. These characteristic broads did not exist in the DBT-removed polymer nanocomposite (Supplementary Fig. S12c). Also, the broad vibration band at about 3450 cm^−1^ in DBT-removed nanocomposite is characteristic of surface O―H stretching vibrations, whereas this band is rather weak in the un-eluted polymer nanocomposite (Supplementary Fig. S12b). This indicates that some DBT molecules interact with carboxylic groups of methacrylic acid through H-bonding. However, after DBT elution, the carboxylic groups of methacrylic acid are released, which might cause a broad vibration band. The cycles of repeated use of SMIP-PMAA@MCM-48-HPW nanocomposite are presented in Supplementary Fig. S13. It was found that the desulfurization efficiency decreased from 96 to 90%, which might be related to the covering of the active oxidation sites of MCM-48-HPW with oxidative products and a slight loss of surface-imprinted polymer in the recycling.

## Conclusions

The results of this study highlight the potential use of surface molecularly imprinted polymer (SMIP) nanocomposites in simultaneous oxidative and adsorptive desulfurization of model and real fuels. We have designed a new oxidative/adsorptive desulfurization system based on an organic–inorganic hybrid SMIP-PMAA@MCM-48-HPW nanocomposite using CCD. A SMIP was prepared on the surface of amine-functionalized silica nanoparticles for the selective recognition and adsorption of DBT by using methacrylic acid as the functional monomer, EGDMA as the crosslinker, and DBT as the template molecules. First, MCM-48-supported catalysts were prepared at different weight percentages of HPW. Different amounts of template were used during the surface polymerization step, and finally, various oxidant/sulfur molar ratios were applied in the desulfurization experiments. The prepared imprinted polymer nanocomposites can interact with DBT molecules through hydrogen bonds, shape selection, and electrostatic interactions. The chemisorbed DBT molecules (by hydrogen bonding) can be oxidized to their corresponding sulfone during the ODS reaction and extracted into acetonitrile phase. Additionally, some DBT molecules can be recognized and captured by the cavities of the molecularly imprinted polymer. Therefore, DBT removal is mainly a combination of trapping of some DBT molecules and oxidizing some DBT molecules that are chemisorbed on the surface sites, resulting in an increased total desulfurization efficiency. The experimental data exhibited excellent DBT removal of 98% at 39.99 wt. % of HPW, 0.096 mmol template amount, and an oxidant/sulfur ratio of 6.94. The reusability tests showed that SMIP-PMAA@MCM-48-HPW could be reused many times without a significant reduction in desulfurization ability. Our results can lead to the development of new systems based on hybrid organic–inorganic nanocomposites with a combined oxidation and adsorption role for efficient desulfurization.

## Methods

### Materials

All the reagents used in the preparation of the polymer nanocomposite materials, namely, Cetyltrimethylammonium Bromide (CTAB, Sigma-Aldrich, > 98% purity), Tetraethylorthosilicate (TEOS, Sigma-Aldrich, 99 wt%), Hydrochloric acid (HCl, Merck, 37 wt%), 12-Tungstophosphoric acid H_3_PW_12_O_40_.xH_2_O (HPW, Sigma-Aldrich, 99.9%), 3-aminopropyl trimethoxysilane (APTMS, Sigma-Aldrich, 97%), Dibenzothiohphene (DBT, Sigma-Aldrich, 98%), Methacrylic acid (MAA, Merck, ≥ 99%) as monomers, Ethylene glycol dimethacrylate (EGDMA, Merck, 98%), Azobis(isobutyronitrile) (AIBN, Merck, > 98%), Acetonitrile (Merck, > 99.9%) and *n*-Heptane (Merck, > 98%) were used as received.

### Synthesis of nanocomposites

#### Synthesis of MCM-48, MCM-48-HPW and amine-functionalized MCM-48-HPW

First, the MCM-48 type of mesoporous silica nanoparticles (MSNs) was synthesized by a facile method at room temperature according to previous literature^30,44^. 12-Tungstophosphoric acid was incorporated into MCM-48 using a direct synthesis method by modifying the MCM-48 synthesis procedure. A homogeneous reaction mixture with a composition of 1TEOS: 12.5NH_4_OH: 54EtOH: xHPW: 0.4CTAB: 174H_2_O was acquired. The weight of the catalyst was determined to be 20–40% of the weight of TEOS, according to the CCD. After aging, washing, and drying the product, the template (CTAB) was removed by calcination at 400 °C for 6 h in the air. The surface of MCM-48-xHPW samples was functionalized by introducing the amino groups of APTMS on the silanols of the silica nanoparticles according to a previous study^24^ to obtain NH_2_-MCM-48-xHPW.

#### Preparation of SMIP

**Figure 7:**
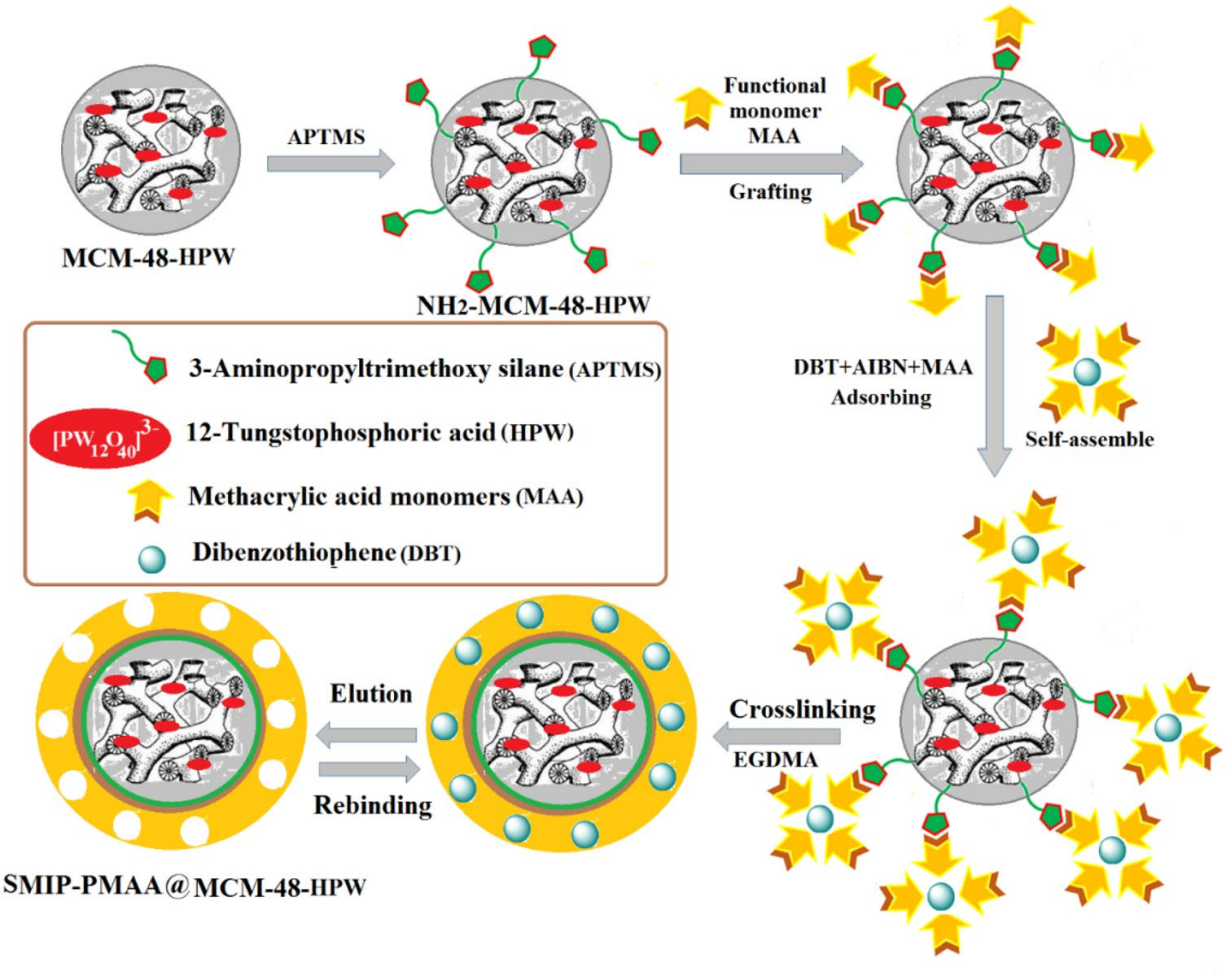
Schematic procedure of SMIP-PMAA@MCM-48-HPW preparation.

First, 1 g of NH_2_-MCM-48-xHPW, 30 ml of CH_2_Cl_2_ and a few drops of pyridine were placed into a 100 ml three-necked flask. After stirring for 20 min, 15 ml of MAA was added slowly, and the mixture was left stirring overnight at 90 °C. After filtering, washing with toluene, acetone and methanol, and drying at 80 °C, the modified NH_2_-MCM-48-xHPW was put into a 500 ml three-neck flask and mixed with 50 ml toluene, 0.45 mmol MAA monomer, desired amounts of DBT (according to the CCD), and 0.018 mmol AIBN as initiator under continuous stirring at 4 °C for 3 h. Then, 1.11 mmol cross-linking agent (EGDMA) was added, and the mixture was purged with N_2_ gas to remove oxygen. Then, the bottom flask was placed into an oil bath pre-heated at 60 °C for 24h. The product was then collected with centrifugation and washed with a methanol/acetic acid (9:1, v/v) solution to remove weakly adsorbed monomers/reactants and template molecules, followed by drying in a vacuum oven at 50 °C for 6 h. The preparation process of polymer nanocomposites is shown in Fig. 7.

#### Desulfurization performance in the oxidative/adsorptive system

For the oxidative/adsorption experiments, first, a 500 ml three-necked glass flask was loaded with model fuel (or gasoline as real fuel oil). The model fuel of 1000 ppm DBT was obtained by dissolving 0.5 g of DBT in 500 ml of *n*-heptane, from which working solutions of 50 ml were prepared to perform the desulfurization process. Then, 0.5 g nanocomposite was added, and the mixture was gently stirred for 2 h to reach the adsorption equilibrium. Then, the ODS process was initiated by adding desired amounts of 30 wt. % H_2_O_2_ (according to the CCD) and 10 ml acetonitrile as extraction solvent to the reaction flask and the biphasic mixture was continuously stirred for 2 h. The reaction temperature was set at 60 °C, according to optimum results obtained from previous studies^45,46^. After the desulfurization, the nanocomposite was separated by filtration, washed with toluene and methanol/acetic acid (9:1) to remove weakly adsorbed copolymer and extract the trapped DBT molecules and dried at 50 °C for 10 h before being subject to the next desulfurization process. The residual concentration of the DBT in the organic phase was determined using HPLC. The desulfurization percentage was calculated according to Eq. (3):

3 $$ {\text{Desulfurization}}\;\left( \% \right) = \left( {\left( {{\text{C}}_{0} {-}{\text{ C}}_{{\text{t}}} } \right)/{\text{C}}_{0} } \right) \times {1}00 $$

where C_0_ and C_t_ present the initial and final concentrations of DBT in the organic phase, respectively.

#### Characterization

The FTIR spectra of prepared materials were recorded on a Bruker Tensor 27 FT-IR spectrometer in the range of 4000–600 cm^−1^ at room temperature. A Bruker D2 phaser diffractometer was used to obtain powder X-ray diffraction patterns in the range of 2θ = 1°–5°. ^1^H NMR spectra of the optimum polymer nanocomposite were collected on a Bruker DRX-300 instrument (300 MHz for 1H). The textural parameters of the different samples were measured by nitrogen adsorption–desorption at 77 K using a BELSORP-mini II (Japan) instrument. The specific surface area (S_BET_) was measured by the Brunauer–Emmett–Teller (BET) equation using adsorption data in the range of P/P_0_ = 0.05–0.25. The total pore volume (V_total_) was measured from the N_2_ adsorption data at a relative pressure of P/P_0_ = 0.99. The t-plot method was applied for the calculation of micropore volume (V_micro_). The mesopore volume of the porous materials (V_meso_) was obtained by subtracting V_micro_ from V_total_. The pore-size distribution was obtained according to the BJH model. SEM images of prepared samples were recorded using a MIRA III TE-SCAN field-emission scanning electron microscope (FESEM) at 5 kV. TEM images were recorded on a Philips 208 s 100 kV microscope. Thermogravimetric analysis (TGA) for determining the thermal degradation of organic compounds was recorded on the TA SDT-Q600 instrument. To determine the total concentration of DBT, the high-performance liquid chromatography (KNAUER HPLC system, Germany) was used. A TCS-2000S ultraviolet fluorescence sulfur analyzer was used to determine the DBT adsorption capacity of the imprinted polymer nanocomposite. An Agilent 6890/HP 5973 inert gas chromatograph/mass spectrometer with a MS detector was applied for gasoline analysis.

### Supplementary Information

Supplementary Information.

## Data Availability

All data generated or analyzed during this study are included in this published article [and its supplementary information file].
