# Supplementary material for: Designing an efficient organic–inorganic hybrid nanocomposite for simultaneous oxidative/adsorptive desulfurization of model and real fuel oils
Source: Sci Rep. 2023 Sep 13;13:15134. doi: 10.1038/s41598-023-42392-8 (PMC10499789; doi:10.1038/s41598-023-42392-8)
Supplement: Supplementary file 1 — Supplementary Information. [file 41598_2023_42392_MOESM1_ESM.docx]

**Designing an efficient organic-inorganic hybrid nanocomposite for simultaneous oxidative/adsorptive desulfurization of model and real fuel oils**

Mina Sadrara [Corresponding Author, Chemistry Department, Faculty of Science, Imam Khomeini International University, Fax number: (009828)33780040 Qazvin, Iran],

Mohammadreza Khanmohammadi Khorrami [Corresponding Author, Chemistry Department, Faculty of Science, Imam Khomeini International University, Fax number: (009828)33780040 Qazvin, Iran]


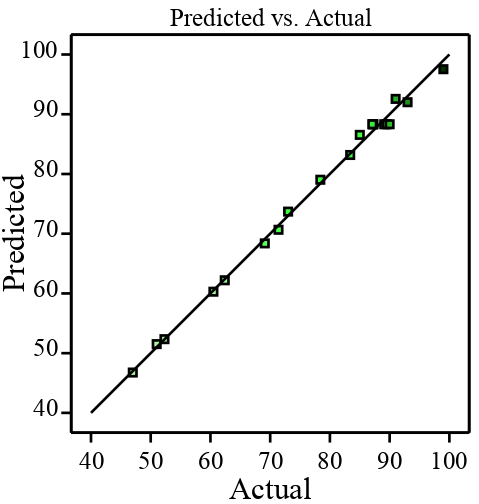


Supplementary Figure S1. The observed values versus the predicted values derived from the model of desulfurization percentage (Y%). The solid line is the regression line with regression coefficient R^2^=0.9959.


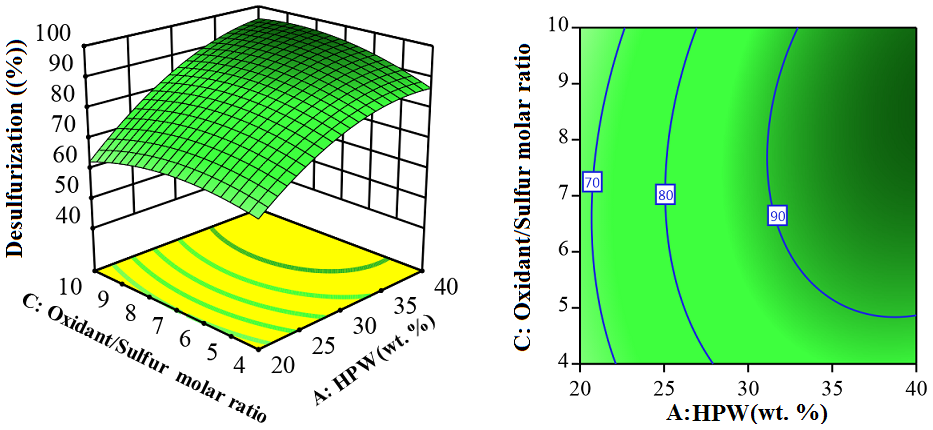


Supplementary Figure S2. Surface (left) and contour (right) plots for the effects of HPW wt. % and oxidant/sulfur molar ratio on the desulfurization percentage


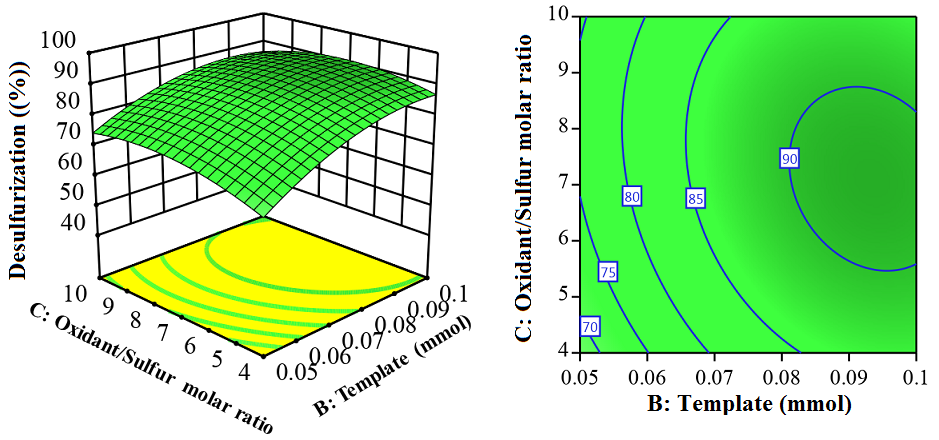


Supplementary Figure S3. Surface (left) and contour (right) plots for the effects of template amount and oxidant/sulfur molar ratio on the desulfurization percentage


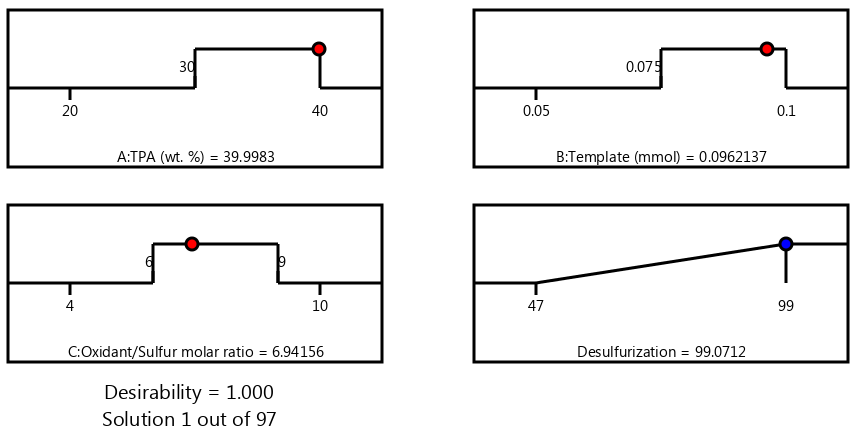


Supplementary Figure S4. First solution of the optimum desulfurization conditions predicted by model using the numerical feature of CCD





Supplementary Figure S5. XRD pattern of MCM-48-HPW, NH_2_-MCM-48-HPW and SMIP-PMAA@MCM-48-HPW





Supplementary Figure S6. N_2_ adsorption-desorption isotherms (a) and the corresponding BJH pore size distribution curves (b) of pure MCM-48, MCM-48-HPW, NH_2_-MCM-48-HPW, SMIP-PMAA@MCM-48-HPW and NIP-PMAA@MCM-48-HPW


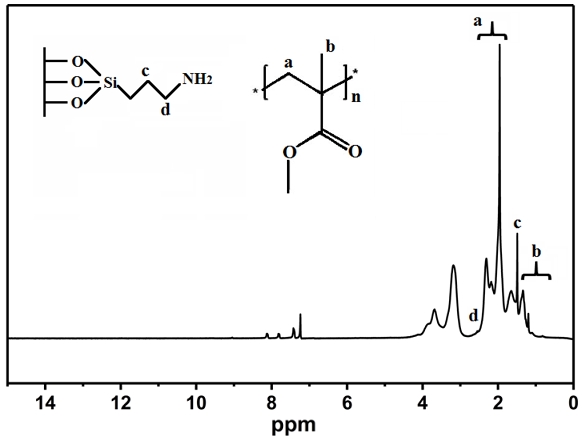


Supplementary Figure S7. ^1^H NMR of SMIP-PMAA@MCM-48-HPW





Supplementary Figure S8. HPLC chromatogram of model fuel before, during and after desulfurization using SMIP-PMAA@MCM-48-HPW nanocomposite





Supplementary Figure S9. HPLC chromatogram of extraction solvent (acetonitrile) at 60, 90 and 120 min desulfurization reaction using optimum SMIP-PMAA@MCM-48-HPW nanocomposite


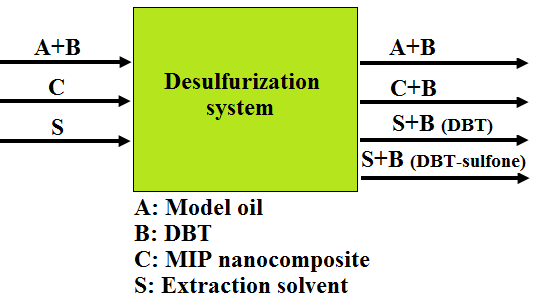


Supplementary Figure S10. Schematic figure of procedure of sulfur mass balance using optimum SMIP-PMAA@MCM-48-HPW nanocomposite


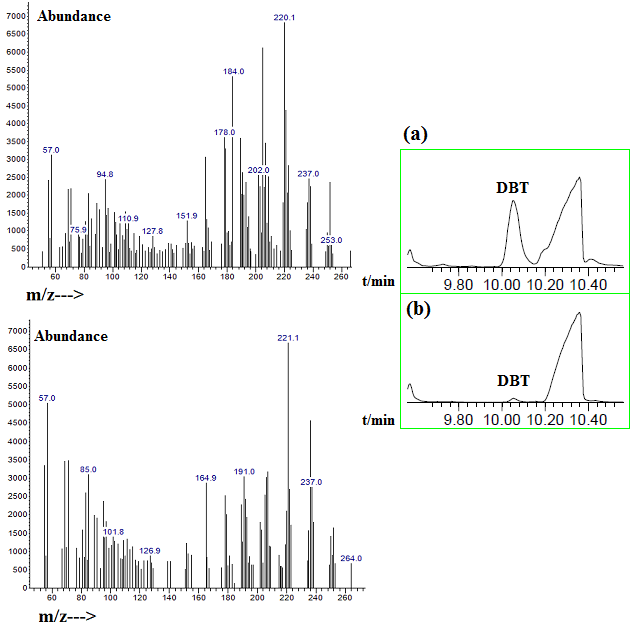


Supplementary Figure S11. GC-MS analysis of gasoline sample before (a, up) and after (b, down) desulfurization


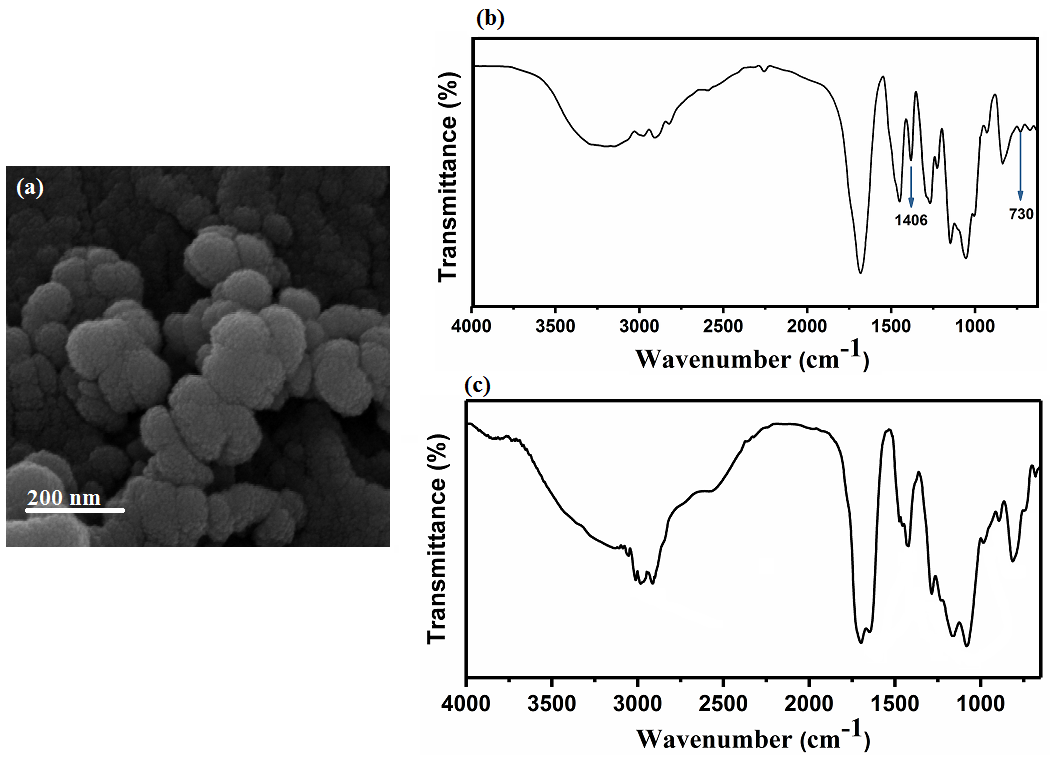


Supplementary Figure S12. Characterization of the spent nanocomposite, SEM image (a), FTIR spectra of spent SMIP-PMAA@MCM-48-HPW before DBT elution (b) and after DBT elution (c).

Supplementary Figure S13. Regeneration performance of polymer nanocomposite in optimum conditions: Catalyst percentage=39.99 wt.%, Template amount: 0.096 mmol, Oxidant/Sulfur molar ratio = 6.941

Supplementary Table S1. Oxidative/adsorptive desulfurization experiments generated by CCD and the corresponding responses.

|  |  | Independent Variables |  |  | Dependent Variable |
| --- | --- | --- | --- | --- | --- |
| Experimental No. | Sample No. | HPW (wt. %) | Template (mmol) | Oxidant/Sulfur molar ratio | Desulfurization (%) |
| 1 | S1 | 0 | 0 | +1.682 | 78.4 |
| 2 | S2 | -1 | -1 | -1 | 51.0 |
| 3 | S3 | +1 | -1 | -1 | 71.4 |
| 4 | S4 | -1 | +1 | +1 | 62.4 |
| 5 | S5 | +1 | +1 | -1 | 93.0 |
| 6 | S6 | -1 | +1 | -1 | 69.1 |
| 7 | S7 | 0 | 0 | 0 | 87.1 |
| 8 | S8 | 0 | 0 | 0 | 89.5 |
| 9 | S9 | +1 | -1 | +1 | 83.4 |
| 10 | S10 | 0 | 0 | 0 | 90.0 |
| 11 | S11 | 0 | 0 | 0 | 87.2 |
| 12 | S12 | 0 | -1.682 | 0 | 60.5 |
| 13 | S13 | -1 | -1 | +1 | 52.3 |
| 14 | S14 | +1.682 | 0 | 0 | 91.0 |
| 15 | S15 | +1 | +1 | +1 | 99.0 |
| 16 | S16 | 0 | +1.682 | 0 | 85.0 |
| 17 | S17 | -1.682 | 0 | 0 | 47.0 |
| 18 | S18 | 0 | 0 | 0 | 89.1 |
| 19 | S19 | 0 | 0 | 0 | 87.3 |
| 20 | S20 | 0 | 0 | -1.682 | 73.0 |

Supplementary Table S2. Lack of fit tests for CCD analysis.

| Source | Sum of Squares | df | Mean Square | f-value | P-value |  |
| --- | --- | --- | --- | --- | --- | --- |
| Linear | 1197.39 | 11 | 108.85 | 63.34 | 0.0001 |  |
| 2FI | 1094.32 | 8 | 136.79 | 79.59 | <0.0001 |  |
| Quadratic | 10.38 | 5 | 2.08 | 1.21 | 0.4204 | Suggested |
| Cubic | 4.45 | 1 | 4.45 | 2.59 | 0.1686 | Aliased |
| Pure Error | 8.59 | 5 | 1.72 |  |  |  |

Supplementary Table S3. Results of model summary statistics analysis for the response.

| Source | Std. Dev. | R^2^ | Adjusted R^2^ | Predicted R^2^ | PRESS | Comments |
| --- | --- | --- | --- | --- | --- | --- |
| Linear | 8.68 | 0.7382 | 0.6891 | 0.6347 | 1682.60 |  |
| 2FI | 9.21 | 0.7605 | 0.6500 | 0.4474 | 2544.98 |  |
| Quadratic | 1.38 | 0.9959 | 0.9922 | 0.9801 | 91.87 | Suggested |
| Cubic | 1.47 | 0.9972 | 0.9910 | 0.7844 | 992.88 | Aliased |

Supplementary Table S4. ANOVA table for the CCD.

| Source | Sum of Squares | df | Mean Square | F-value | p-value |  |
| --- | --- | --- | --- | --- | --- | --- |
| Model | 4586.77 | 9 | 509.64 | 268.62 | < 0.0001 | significant |
| A-HPW (wt. %) | 2533.20 | 1 | 2533.20 | 1335.18 | < 0.0001 |  |
| B-Template (mmol) | 832.14 | 1 | 832.14 | 438.60 | < 0.0001 |  |
| C-Oxidant/Sulfur molar ratio | 34.42 | 1 | 34.42 | 18.14 | 0.0017 |  |
| AB | 10.13 | 1 | 10.13 | 5.34 | 0.0435 |  |
| AC | 68.45 | 1 | 68.45 | 36.08 | 0.0001 |  |
| BC | 24.50 | 1 | 24.50 | 12.91 | 0.0049 |  |
| A² | 627.40 | 1 | 627.40 | 330.68 | < 0.0001 |  |
| B² | 400.59 | 1 | 400.59 | 211.14 | < 0.0001 |  |
| C² | 257.77 | 1 | 257.77 | 135.87 | < 0.0001 |  |
| Residual | 18.97 | 10 | 1.90 |  |  |  |
| Lack of Fit | 10.38 | 5 | 2.08 | 1.21 | 0.4204 | not significant |
| Pure Error | 8.59 | 5 | 1.72 |  |  |  |
| Cor Total | 4605.75 | 19 |  |  |  |  |
| R^2^=0.9959 |  |  |  |  |  |  |
| Adjusted R^2^=0.9922 |  |  |  |  |  |  |
| Predicted R^2^=0.9801 |  |  |  |  |  |  |

Supplementary Table S5. Limits used to obtain the maximum value for the desulfurization percentage.

| Name | Goal | Lower limit | Upper limit | Importance |
| --- | --- | --- | --- | --- |
| A:HPW percentage (wt. %) | In range | 30 | 40 | 3 |
| B:Template amount (mmol) | In range | 0.075 | 0.1 | 3 |
| C:Oxidant/Sulfur molar ratio | In range | 6 | 9 | 3 |
| Y:Desulfurization (%) | Maximize | 47 | 99 | 3 |

Supplementary Table S6. Mass balance for 1000 ppm DBT in desulfurization process

| Input | Mass (ppm/g) | Process | Output | Measured mass (g) | Balance (g) | % |
| --- | --- | --- | --- | --- | --- | --- |
|  |  |  | DBT trapped in nanocomposite | 1.524×10^-2^ g DBT |  | 30.48 |
| DBT in model oil (initial concentration) | 1000 ppm DBT or  0.05 g DBT in 50 ml model oil | Oxidative/adsorptive desulfurization | DBT-sulfone extracted into acetonitrile | 1.599×10^-4^ mol DBT-Sulfone in acetonitrile phase  equivalent to 1.599×10^-4^ mol DBT (2.947×10^-2^ g) | 4.8195×10^-2^ g DBT | 58.94 |
|  |  |  | DBT extracted into acetonitrile | 2.755×10^-3^ g DBT |  | 5.51 |
|  |  |  | Remaining DBT in model oil | 7.300×10^-4^ g DBT |  | 1.46 |
|  |  |  | Waste (not detected) | 1.805×10^-3^ g DBT | 1.8050×10^-3^ | 3.61 |
